# Hematological markers of proinflammatory and prothrombotic states in normal-weight obesity (NWO).

**DOI:** 10.1038/s41598-026-48634-9

**Published:** 2026-04-28

**Authors:** Waldemar Pluta, Anna Lubkowska, Wioleta Dudzińska

**Affiliations:** 1 Department of Functional Diagnostics and Physical Medicine, Pomeranian Medical University in Szczecin, Żołnierska 54, Szczecin, 71-210 Poland; 2 Institute of Biology, University of Szczecin, Felczaka 3c, Szczecin, 71-412 Poland

**Keywords:** Normal weight obesity, Hematological patterns, DXA, Percent body fat, Proinflammatory and prothrombotic state, Biomarkers, Diseases, Endocrinology, Medical research, Physiology

## Abstract

Normal Weight Obesity (NWO) is characterized by excessive body fat despite a normal BMI. This study aimed to assess whether young women with the NWO phenotype exhibit a different hematological profile compared with their peers with a normal body composition. The study included 176 young women aged 18–24 years with a normal BMI (18.5–24.9 kg/m²). Based on a previously established cutoff point for percentage body fat (PBF), participants were assigned to the NWO group (PBF ≥ 35.8%) and the control group (PBF < 35.8%). Complete blood counts and biochemical parameters were analyzed. It was demonstrated that women in the NWO group had significantly higher leukocyte, lymphocyte, and platelet counts compared with the control group. Significantly higher plateletcrit and large platelet counts, as well as higher hs-CRP levels, were also observed. Significant positive correlations were found between PBF and the hematological parameters examined. Based on the results, the NWO phenotype in young, clinically healthy women is associated with a subclinical proinflammatory state and prothrombotic potential. Routine hematological parameters may be a cost-effective, readily available tool for early assessment of cardiometabolic risk in individuals with a normal body weight.

**Supplementary Information:**

The online version contains supplementary material available at 10.1038/s41598-026-48634-9.

## Introduction

Obesity and its associated metabolic disorders constitute a rapidly escalating global health challenge. While Body Mass Index (BMI) remains the traditional standard for weight classification, its diagnostic limitations are increasingly recognized – specifically its inability to distinguish between fat-free mass and adipose tissue. Consequently, reliance on BMI alone may lead to the underestimation of cardiometabolic risk, particularly in individuals presenting with the NWO phenotype. This specific condition, characterized by excess body fat accumulation despite a normal BMI, represents a “hidden” form of obesity associated with a significantly elevated risk of metabolic dysregulation^1^.

Adipose tissue is no longer viewed only as an energy reservoir but as a dynamic endocrine organ^2^. In states of excess adiposity, such as NWO, adipose tissue becomes dysfunctional and secretes proinflammatory cytokines (e.g., IL-6, TNF-α) and adipokines. This low-grade chronic inflammation is a key mechanism linking obesity to cardiovascular disease^3^. Importantly, systemic inflammation directly influences hematopoiesis in the bone marrow, potentially leading to alterations in peripheral blood cell counts^4^. Therefore, hematological parameters - widely available through routine Complete Blood Count (CBC) tests - could serve as early, cost-effective biomarkers of the metabolic dysregulation associated with NWO.

White blood cell (WBC) count is a well-established marker of systemic inflammation and an independent predictor of cardiovascular events^5,6^ and type 2 diabetes^7^. Similarly, platelet indices, such as Mean Platelet Volume (MPV) and the increasingly recognized Platelet Large Cell Count (P-LCC), reflect platelet activation and thrombotic potential^8^. Despite this, research specifically analyzing the relationship between detailed body composition and these hematological markers in young, apparently healthy women remains limited. Most studies focus on older populations or individuals with diagnosed metabolic syndrome^9,10^, leaving a gap in our understanding of early subclinical changes in the NWO phenotype.

This study is a continuation of our previous research, in which we focused on the analysis of body composition and metabolic parameters in young women (18–24 years old) from northwestern Poland who had a normal BMI (18.5–24.9 kg/m²). Using dual-energy X-ray absorptiometry (DXA), we identified the phenotype of metabolic obesity with normal body weight (NWO) in 27.3% of the studied women. The key achievement was establishing a new cutoff for PBF at 35.8%, effectively identifying individuals with increased cardiometabolic risk. Women with the NWO phenotype were characterized by higher insulin values (13.4 vs. 10.4 µIU/ml), HOMA-IR index (2.3 vs. 1.7), and an unfavorable lipid profile, including increased LDL cholesterol (2.1 vs. 1.6 mM), triglycerides (0.8 vs. 0.6 mM), and decreased HDL.

(1.1 vs. 1.2 mM) compared with the control group. A particularly important finding was the android-to-gynoid adipose tissue distribution ratio (A/G), which showed correlations with insulin resistance (*r* = 0.21) and HDL concentration (*r* = -0.32)^1^.

The present study aimed to evaluate whether young women who meet this NWO criterion exhibit distinct hematological profiles compared with their lean peers. Specifically, we investigated the association among PBF, lipid profile, and a comprehensive panel of hematological indices - including inflammatory (WBC, lymphocytes) and thrombotic markers (PLT, P-LCC) - to determine their utility in early cardiometabolic risk stratification.

## Materials and methods

The study was conducted in accordance with the principles of the Declaration of Helsinki and with prior approval from the Bioethics Committee of the Pomeranian Medical University in Szczecin.

(no. KB-0012/13/2021). Written informed consent was obtained from all participants.

### Volunteers

This study is a continuation of our previous project^1^, in which we established a PBF cutoff to identify young women with normal BMI who are at increased cardiometabolic risk.

Participants were assigned to groups based on a PBF cutoff of 35.78% (rounded to 35.8% based on a statistically calculated value). Eighty-eight individuals were selected for the NWO group (BMI 18.5–24.9 kg/m², PBF ≥ 35.8%) and 88 individuals for the control group (normal weight; BMI 18.5–24.9 kg/m²,

PBF < 35.8%) from the original volunteer cohort. To minimize selection bias, participants from the original cohort were matched 1:1 to the NWO and control groups, ensuring homogeneity of both groups with respect to age and BMI.

### Research procedures

#### Anthropometric measurements

The anthropometric study protocol consisted of measuring:

1. height and weight – using a Seca 711 stadiometer (accuracy of 0.01 kg and 1 mm),
2. waist circumference (WC) and hip circumference (HC) – using a Seca 203 anthropometric tape (accuracy of 1 mm).

WC was measured halfway between the lower edge of the rib and the iliac process, while HC was measured above the buttocks at the widest point around the greater trochanter. During all measurements, the volunteers wore only underwear. Each measurement was taken twice, and the arithmetic mean was used for further analyses. Based on the obtained results, body mass index (BMI), waist-to-hip ratio (WHR), and waist-to-height ratio (WHtR) were calculated.

Anthropometric data were retrieved from our existing database described in our previous manuscript^1^; no new measurements were performed for this specific analysis.

#### Body composition analysis

Body composition analysis was performed using dual-energy X-ray absorptiometry (DXA) with the Hologic device QDR 4500 W^®^ (Quirugil, Bogota, Colombia) and Hologic APEX software (APEX Version 5.6.0.4, Hologic Inc., Marlborough, MA, USA; https://www.hologic.com), using a high-resolution whole-body scan. According to the manufacturer, the device is characterized by the following values of measurement repeatability and precision: (1) intraclass correlation coefficients for total fat mass, percentage of body fat, and lean soft tissue: 0.997–0.999; (2) coefficient of variation for fat mass: 1.2%; for lean mass: 1.1%. Daily calibration using a spine phantom, according to the manufacturer’s recommendations, ensured the reliability and high quality of the results. The examination was performed according to the manufacturer’s recommendations: the patient was positioned supine, with arms alongside the body, thumbs on the iliac crest, remaining fingers spread, and legs internally rotated by 25 degrees. The patient lay still and breathed normally during the examination. To eliminate human error and ensure reliable results, scans were performed and analyzed by a single, trained technician.

Body composition results were retrieved from our existing database described in our previous manuscript^1^; no new measurements were performed for this specific analysis.

#### Blood collection and analysis of hematological parameters

Blood was collected from patients after an overnight fast, between 6:00 AM and 8:00 AM, after a 10-minute rest in a sitting position, by qualified medical personnel from the basilic vein, in accordance with applicable standards, using a Vacutainer^®^ vacuum suction device (Vacutainer System, BD-Plymouth, PL6 7BP, United Kingdom). A total volume of 15 ml of blood was collected into tubes containing EDTA-K2.

(to obtain plasma) and tubes with a clot activator spray (to obtain serum). The blood was centrifuged and aliquoted according to applicable procedures and stored at -70 °C in an Eppendorf^®^ CryoCube^®^ F570 freezer until further analysis.

Similar to anthropometric measurements and body composition analysis, hematological data were retrieved from the dataset obtained in the first stage of the project^1^.

Hematological parameters were measured in whole blood using a BM HEM-3 hematology analyzer (Biomaxima S.A., Lublin, Poland). To ensure measurement accuracy and precision, the analyzer was calibrated daily and underwent regular quality control procedures using reference materials, as per the manufacturer’s protocol. All laboratory procedures were conducted by trained personnel to ensure technical accuracy and reproducibility of the results. The following parameters were examined: white blood cell count (WBC), red blood cell count (RBC), hemoglobin (HGB), hematocrit (HCT), mean corpuscular volume (MCV), mean corpuscular hemoglobin mass (MCH), mean corpuscular hemoglobin concentration (MCHC), platelets (PLT), plateletcrit (PCT), mean platelet volume (MPV), platelet distribution width (PDWs, PDWc), red blood cell distribution width (RDWs, RDWc), lymphocytes (LYM), medium-sized cells (MID), granulocytes (GRA), percentage of lymphocytes (LYM%), percentage of medium-sized cells (MID%), percentage of granulocytes (GRA%), number of large platelets (P-LCC) and large platelet index (P-LCR).

The detailed methodology and recruitment process were described in the previous work^1^.

### Statistical analysis

The minimal size of the study sample was estimated using the G*Power 3.1.9.4 software. The effect size was set at 0.5, the error probability α was 0.05, and the power was 0.95. The non-central parameter δ was 3.317. The total sample size was 176 and the actual power was 0.951.

Statistical analysis was performed using Statistica 13.3 (Statistica PL, StatSoft, Krakow, Poland) software. The Shapiro-Wilk test was used to check the normality of the distribution. Since the data distribution was not normal, nonparametric tests were used for detailed statistical analyses. Results were presented as median and interquartile range (25%-75%; Q25-Q75). The Mann-Whitney U test was used to show the significance of differences. Spearman correlation coefficients were used to describe the relationships between continuous variables.

Due to the exploratory nature of this study, which aimed to identify a broad range of hematological deviations in the NWO phenotype, no formal correction for multiple comparisons was applied. This approach was chosen to minimize the risk of Type II errors and to preserve the sensitivity of the analysis for identifying subtle subclinical associations, which were further validated by their biological consistency across multiple parameters.

The level of significance was set at *p* < 0.05.

## Results

### Group characteristics

The median age was identical in both groups (20 years). Women in the NWO group exhibited significantly higher anthropometric values compared with the control group. Body weight was significantly higher (62 vs. 58 kg, *p* < 0.0001), as were waist circumference (WC) (73 vs. 69 cm, *p* < 0.0001), hip circumference (HC) (99 vs. 94 cm, *p* < 0.0001), and percentage body fat (PBF) (37.9 vs. 32.1%, *p* < 0.0001). Body mass index (BMI) (22.7 vs. 20.4 kg/m², *p* < 0.0001) and waist-to-height ratio (WHtR) (43 vs. 41,

*p* < 0.0001) were also significantly higher in the NWO group. Detailed values of the analyzed anthropometric parameters are presented in Table 1.

**Table 1:** Anthropometric characteristics of the subjects.

	All (*n* = 176)		NW (*n* = 88)		NWO (*n* = 88)		*P*-value
	median	Q_25_-Q_75_	median	Q_25_-Q_75_	median	Q_25_-Q_75_	
Age [years]	**20**	19–22	**20**	19–22	**20**	19–22	0.500
Weight [kg]	**60**	55–65	**58**	54–63	**62**	57–67	*p* < 0.0001
Height [cm]	**168**	163–172	**169**	164–173	**167**	163–172	0.132
BMI [kg/m^2^]	**21.4**	20.2–23.1	**20.4**	19.5–21.7	**22.7**	21.3–23.7	*p* < 0.0001
PBF [%]	**35.2**	32.1–38	**32.1**	29.5–33.4	**37.9**	36.7–39.4	*p* < 0.0001
WC [cm]	**70**	67–74	**69**	66–73	**73**	69–75	*p* < 0.0001
HC [cm]	**97**	91–100	**94**	90–97	**99**	96–103	*p* < 0.0001
WHR	**0.7**	0.70–0.76	**0.7**	0.71–0.76	**0.7**	0.69–0.76	0.550
WHtR	**42**	40–44	**41**	39–43	**43**	41–45	*p* < 0.0001

BMI, body mass index; PBF, percent body fat; WC, waist circumference; HC, hip circumference; WHR, waist to hip ratio; WHtR, waist to height ratio.

Analysis of body composition revealed that women in the NWO group had significantly higher body fat content. These differences were evident in both relative (percent body fat (PBF), fat mass (FM), appendicular fat mass (AFM) and absolute (fat mass index (FMI), appendicular fat mass ratio (AFMI)) values of fatness indices (*p* < 0.001 for all comparisons). A higher fat mass ratio (FMR) further confirmed increased fat accumulation in the NWO group (*p* < 0.001). The android to gynoid adipose tissue ratio (A/G) was significantly higher in women in the NWO group (*p* < 0.001). Analysis of visceral adipose tissue deposition, expressed by visceral fat area (VFA), mass (VFM), and volume (VFV), showed significantly higher values in the NWO group (*p* < 0.001 for all parameters). Analysis of fat-free deposit showed lower values for all indices in the NWO group, with only appendicular fat-free mass (AFFM) and appendicular lean mass (ALM) were statistically significantly lower (*p* < 0.05) compared with the control group. Detailed values for body composition parameters are presented in the supplementary material (**Table ****S1****.**).

The analysis of hematological parameters showed that the control group, compared with the NWO group, was characterized by higher, but statistically non-significant (*p* > 0.05) values of: red blood cell distribution width (CV) (RDWc) (15.6 vs. 15.3%), medium-sized cells (MID) (0.34 vs. 0.31 × 10^9^/l), percentage of medium-sized cells (MID%) (6 vs. 5.7%) and percentage of granulocytes (GRA%).

1. In contrast, the NWO group had higher, but statistically non-significant (*p* > 0.05) values of: red blood cells count (RBC) (4.6 vs. 4.5 × 10^9^/l), hemoglobin (HGB) (128.3 vs. 124.5 g/l), hematocrit (HCT) (37.4 vs. 36.5%), mean corpuscular volume (MCV) (84 vs. 81 fl.), mean corpuscular hemoglobin mass (MCH) (28.7 vs. 28 pg), mean corpuscular hemoglobin concentration (MCHC) (342 vs. 340 g/l), mean platelet volume (MPV) (8.8 vs. 8.1 fl.), platelet distribution width (SD) (PDWs) (11.8 vs. 11.1 fl.), platelet distribution width (CV) (PDWc) (38.5 vs. 37.7%), red cell distribution width (SD) (RDWs).
2. Granulocytes (GRA) (3.7 vs. 3.3 × 10^9^/l), lymphocyte percentage (LYM%).
3. Platelet large cell ratio (P-LCR) (27.7 vs. 25.4%). Among the remaining parameters, significantly higher values in the NWO group compared to the control group were observed for white blood cells (WBC) (6.3 vs. 5.7 × 10^9^/l; *p* = 0.045), plateletcrit (PCT) (0.21 vs. 0.18%; *p* = 0.001), lymphocytes (LYM) (2.1 vs. 1.8 × 10^9^/l; *p* = 0.005), and the number of large platelets (P-LCC).

(67 vs. 57 × 10^9^/l; *p* = 0.003). In addition, the NWO group was characterized by a significantly higher platelet count (PLT) (244 vs. 222 × 10^9^/l; *p* = 0.010). The NWO group was characterized by a significantly higher high-sensitivity C-reactive protein (hs-CRP) concentration (0.63 vs. 0.6 mg/l; *p* = 0.014) with a lower (but not statistically significant) nitric oxide (NO) concentration (6.44 vs. 7.72 µM; *p* = 0.479) compared to the control group. Detailed values of the analysis of hematological and biochemical parameters are presented in Table 2.

**Table 2:** Hematological and biochemical parameters of study participants.

	All (*n* = 176)		NW (*n* = 88)		NWO (*n* = 88)		*P*-value
	median	Q_25_-Q_75_	median	Q_25_–Q_75_	median	Q_25_–Q_75_	
WBC [x 10^9^/l]	6.1	5-7.3	5.7	4.8-6.0	6.3	5.3–7.5	0.045
RBC [x 10^9^/l]	4.5	4.3–4.8	4.5	4.3–4.8	4.6	4.3–4.9	0.331
HGB [g/l]	126.5	119–133	124.5	118–131	128.3	120-134.8	0.112
HCT [%]	36.9	35.4–39	36.5	35.3–38.6	37.4	35.4–39.6	0.069
MCV [fl]	83	77–86	81	76-85.5	84	78-86.5	0.075
MCH [pg]	28.5	26.6–29.3	28	26.1–29.2	28.7	27-29.8	0.112
MCHC [g/l]	341	334–349	340	333–348	342	336.3-349.5	0.664
PLT [x 10^9^/l]	244	199–279	222	185.5–265	244	199–279	0.010
PCT [%]	0.19	0.17–0.22	0.18	0.16–0.2	0.21	0.17–0.26	0.001
MPV [fl]	8.5	7.1–9.8	8.1	7-9.8	8.8	7.4–9.9	0.093
PDWs [fl]	11.4	9.2–13.9	11.1	8.7–13	11.8	9.6–14	0.091
PDWc [%]	38	37.2–39.9	37.7	36.1–39.8	38.5	37.3–40	0.092
RDWs [fl]	39.6	35.4–42.6	39.1	35.4–42.2	39.6	36-42.7	0.919
RDWc [%]	15.4	14.6–16.2	15.6	14.6–16.6	15.3	14.5–15.8	0.160
LYM [x 10^9^/l]	2.04	1.6–2.4	1.8	1.5–2.3	2.1	1.8–2.7	0.005
MID [x 10^9^/l]	0.32	0.21–0.48	0.34	0.21–0.45	0.31	0.21–0.53	0.964
GRA [x 10^9^/l]	3.6	2.8–4.5	3.3	2.7–4.6	3.7	3.1–4.5	0.169
LYM% [%]	34.6	26.5–40.5	31.1	25-39.6	36.3	28.7–40.8	0.059
MID% [%]	5.8	3.7–8.5	6	3.8–8.9	5.7	3.7–7.5	0.341
GRA% [%]	59.7	53-67.5	61.5	53.4–67.8	57.8	52.6–66.5	0.329
P-LCC [x 10^9^/l]	61	49–77	57	44.5–68.5	67	54.8–85.5	0.003
P-LCR [%]	26.3	22.2–34.4	25.4	20.6–33.9	27.7	22.8–34.7	0.180
hs-CRP [mg/l]	0.6	0.6–1.1	0.6	0.6-1	0.63	0.6–1.2	0.014
NO [µM]	7.3	4.6–12.3	7.7	4.6–13.8	6.4	4.8–11.9	0.479

WBC, white blood cells count; RBC, red blood cells count; HGB, hemoglobin; HCT, hematocrit; MCV, mean corpuscular volume; MCH, mean corpuscular hemoglobin mass; MCHC, mean corpuscular hemoglobin concentration; PLT, platelets; PCT, plateletcrit; MPV, mean platelet volume; PDWs, platelet distribution width (SD); PDWc, platelet distribution width (CV); RDWs, red cell distribution width (SD); RDWc, red cell distribution width (CV); LYM, lymphocytes; MID, mid-sized cells; GRA, granulocytes; LYM%, lymphocyte percentage; MID%, mid-sized cell percentage; GRA%, granulocyte percentage; P-LCC, platelet large cell count; P-LCR, platelet large cell ratio; hs-CRP, high-sensitivity C-reactive protein; NO, nitric oxide.

### Correlation analysis

Correlation analysis showed a statistically significant weak, positive correlation between WBC and PBF, TG/G (*r* = 0.20), and TG (*r* = 0.21) (*p* < 0.05).

PCT correlated weakly, positively with HDL-C, HOMA-IR (*r* = 0.22), LAP (*r* = 0.20) (*p* < 0.05), PBF, Non-HDL (*r* = 0.25), TC, glucose (*r* = 0.27), LDL-C (*r* = 0.24), TG (*r* = 0.28), TG/G (*r* = 0.29).

(*p* < 0.01).

In the case of LYM, a weak positive correlation was shown with PBF (*r* = 0.21), TG (*r* = 0.22), TG/G (*r* = 0.20) (*p* < 0.05), LDL-C, Non-HDL (*r* = 0.24), TC (*r* = 0.27), and HDL-C (*r* = 0.25) (*p* < 0.01).

P-LCC statistically significantly correlated with PBF, HOMA-IR (*r* = 0.21), LAP (*r* = 0.20).

(*p* < 0.05), with HDL-C (*r* = 0.30), LDL-C (*r* = 0.27), Non-HDL (*r* = 0.28), TG (*r* = 0.29), TG/G (*r* = 0.31) (*p* < 0.01) and with TC, glucose (*r* = 0.32 *p* < 0.001).

Detailed results of the correlation analysis are presented in Table 3 and in the supplementary material **(Table S2.).**

**Table 3:** Correlations between hematological parameters and percent body fat, lipid profile, and cardiometabolic risk indices.

		WBC	PCT	LYM	*P*-LCC
PBF [%]		0.20*	0.25**	0.21*	0.21*
TC [mM]		0.13	0.27**	0.27**	0.32***
HDL-C [mM]		0.10	0.22*	0.25**	0.30**
LDL-C [mM]		0.11	0.24**	0.24**	0.27**
Non-HDL [mM]		0.13	0.25**	0.24**	0.28**
TG [mM]		0.21*	0.28**	0.22*	0.29**
Glucose [mM]		0.11	0.27**	0.14	0.32***
Insulin [µlU/ml]		-0.01	0.12	0.02	0.07
HOMA-IR		0.10	0.22*	0.08	0.21*
TG/HDL-C		0.08	0.09	-0.02	0.02
TG/G		0.20*	0.29**	0.20*	0.31**
VAI		0.05	0.05	-0.09	-0.01
LAP		0.13	0.20*	0.05	0.20*
CMI		0.08	0.09	-0.06	0.02

WBC, white blood cells count; PLT, platelets; PCT, plateletcrit; LYM, lymphocytes; P-LCC, platelet large cell count; PBF, percent body fat; TC, total cholesterol; HDL-C, high-density lipoprotein cholesterol; LDL-C, low-density lipoprotein cholesterol; Non-HDL, non-high-density lipoprotein cholesterol; TG, triglycerides; HOMA-IR, homeostatic model assessment for insulin resistance; TG/HDL-C, triglycerides to HDL-C ratio; TG/G, triglycerides to glucose ratio; VAI, visceral adiposity index; LAP, lipid accumulation product; CMI, cardiometabolic index; * *p* < 0.05, ** *p* < 0.01, *** *p* < 0.001.

## Discussion

There is evidence of a link between NWO phenotype and an abnormal cardiometabolic profile, both in the international literature^11,12^ and in our previous research^1^. In our prior study, we established a percent body fat (PBF) cutoff point of 35.78% to effectively identify young women with normal BMI who are at increased cardiometabolic risk. Our findings revealed that women with the NWO phenotype exhibited significantly higher fasting insulin levels (13.4 vs. 10.4 µU/ml) and higher HOMA-IR scores (2.3 vs. 1.7) than their lean peers. Furthermore, the NWO group exhibited an unfavorable lipid profile, including elevated levels of total cholesterol, LDL-C, non-HDL-C, and triglycerides, as well as significantly reduced HDL-C. Additionally, the android-to-gynoid (A/G) adipose tissue ratio emerged as a significant predictor of metabolic health, showing a positive correlation with insulin resistance and a strong negative correlation with HDL-C levels. These metabolic disturbances underscore the systemic impact of “hidden” adiposity even in individuals with a normal BMI.

The current study, representative of the same population of young women with NWO obesity, showed that higher PBF levels were associated with higher levels of hematological parameters, including WBC, LYM, PCT, and P-LCC. Our results indicate that, in young women, despite having a normal body weight (as determined by BMI), hematological changes are observed that, when measured in a commonly used and widely available manner, may be associated with the NWO phenotype, associated subclinical proinflammatory state, and increased risk of thrombosis.

Among many hematological indicators, WBC and LYM counts are widely used to diagnose systemic inflammation^13^. It is elevated in obesity, is significantly correlated with insulin resistance, strongly predicts the development of type 2 diabetes in obese and non-obese individuals, metabolic syndrome, and is a predictor of mortality from coronary heart disease, regardless of traditional risk factors for cardiovascular disease^4,10,14–17^. It has also been shown that a high WBC count can predict future cardiovascular events in healthy individuals^18^. In this study, we found for the first time that WBC and LYM counts were significantly higher in women with NWO (6.3 vs. 5.7 × 10^9^/l, *p* = 0.045 and 2.1 vs. 1.8 × 10^9^/l, *p* = 0.005, respectively).

4. While the mean values did not exceed the accepted physiological range, there was relative leukocytosis and lymphocytosis, with a progressive increase dependent on PBF. This suggests that the observed changes may be mediated in part by an increase in adipose tissue content and the release of inflammatory markers from it. The association between inflammation and NWO has been highlighted in several studies and, more recently, summarized in a systematic review and meta-analysis^19^. The presence of a subclinical proinflammatory state in the NWO phenotype was primarily associated with elevated IL-6 and CRP levels, potent inducers of leukocytosis^4^. Our current study confirms these previous findings. We observed a significant increase in hs-CRP levels in the NWO group.
5. Although hs-CRP levels were within the reference ranges in both groups, a significant difference remained between them. Previous studies have shown that concomitant exposure to elevated levels of proinflammatory markers, such as CRP and WBC, correlated with the development of several disease states in obese individuals^20–22^. Our study extends these findings to young non-obese women, which has important implications for their health risks. Interestingly, PBF is associated with.

hs-CRP and WBC counts independently of BMI. These changes cannot be explained by factors or diseases that typically increase CRP and WBC counts. We observed these associations in healthy, very young women, so subclinical disease is unlikely to explain our results. These data indicate a potential subclinical proinflammatory state in young women with the NWO phenotype, as evidenced by increased WBC counts and hs-CRP.

Elevated hs-CRP levels within the reference range in apparently healthy men and women have been associated with an increased risk of developing cardiovascular disease, diabetes, and nonalcoholic fatty liver disease^23–25^. Numerous clinical studies have linked CRP with an increased risk of myocardial infarction, stroke, peripheral artery disease, and sudden death^26^. Furthermore, elevated CRP levels are one of the strongest predictors of progressive vascular disease^27^. Moreover, CRP can activate the endothelium by modulating the endothelial nitric oxide synthase-nitric oxide pathway. Elevated CRP levels are associated with a significant decrease in NO production, which is a consequence of CRP’s action to reduce endothelial nitric oxide synthase mRNA stability and protein levels^28,29^. In our group of women with NWO, we observed a non-significantly lower plasma NO concentration than in the control group (6.44 vs. 7.72 µM; *p* = 0.479), consistent with extensive literature describing reduced NO bioavailability in obesity^30–33^. Such endothelial dysfunction promotes the activation and adhesion of PLTs and WBCs, as well as the release of proinflammatory cytokines. These phenomena increase vascular permeability to oxidized lipoproteins and inflammatory mediators, ultimately causing structural changes in the arterial wall, including smooth muscle cell proliferation and the initiation of atherosclerotic plaque development^34^. Platelet activation is also increased in NO-deficient states, contributing to thrombosis and increasing the risk of acute cardiovascular events such as myocardial infarction or stroke^35^.

The results of this study provided another interesting observation. It concerns the association of WBC and LYM counts with several cardiovascular risk factors, including TG levels and the TG/G ratio. This is consistent with the findings of Ohshita et al.^32^ and Targher et al.^36^. They reported that in individuals with normal fasting glucose, an elevated, yet normal, WBC count is associated with a set of metabolic abnormalities typical of the insulin resistance syndrome and is associated with some components of the metabolic syndrome, such as BMI, WHR, and TG levels. The impact of TG on cardiovascular risk has long been well documented^37^. It is also known that combined exposure to high WBC counts and TG levels that are consistently within the reference range is associated with a more than threefold increased risk of cardiovascular mortality, independent of traditional risk factors^38^. In our analysis, WBC count and previously assessed TG concentration (0.8 vs. 0.6 mM; *p* = 0.01;^1^ were significantly higher in the NWO group, and these variables were associated. These data provide new evidence suggesting that combined exposure to high TG concentrations and WBC counts may be associated with an increased risk of cardiovascular disease in young women with the NWO phenotype. This finding is plausible in our cohort. TG concentration has been shown to be closely associated with inflammation, including elevated WBC counts^39^. This association is thought to occur through indirect mediators such as adipose tissue or insulin resistance^40,41^. Our group of women with NWO has increased PBF and is insulin resistant (HOMA-IR: 2.3 vs. 1.7; *p* = 0.002)^1^. Therefore, the results may be consistent with the hypothesis that individuals with high WBC counts and high TG concentrations are more insulin-resistant, which further increases their cardiovascular risk.

One result of this study is a significant increase in PLT, P-LCC, and PCT counts in women with NWO. We also found positive associations between PLT and P-LCC counts and between PLT and PBF. Although PLT counts are important for quantifying the coagulation process, they are significantly higher in obesity and metabolic syndrome^8,33,42^. They are also associated with the risk of arterial and venous thrombosis in obese individuals^4^ and the risk of type 2 diabetes^43^. Nevertheless, we believe that, in addition to PLT and PCT, the importance of markers that measure the number of larger, more active platelets should be emphasized. P-LCC is such an indicator. We found no other studies in the literature that assessed P-LCC in women with NWO. In our research, we found that P-LCC was significantly higher in this group of subjects (67 vs. 57 × 10^9^/l; *p* = 0.003). This result was also consistent with significantly higher PLT counts (244 vs. 222 × 10^9^/l; *p* = 0.010). Larger platelets are more reactive and have a greater ability to form clots, increasing the risk of thrombosis^44^. They are considered immature, and their numbers increase with factors that influence platelet turnover. Inflammation is considered the most important factor for increased platelet turnover^8,45,46^. Exposure to inflammatory signals can accelerate the production of larger, more reactive PLTs^47^. We hypothesize that, in women with NWO, inflammation may lead to a subclinical increase in platelet formation and a corresponding acceleration of their entry into peripheral blood, which may explain the high PLT and P-LCC counts in our study. Furthermore, PCT, which measures the total platelet volume as a percentage of the volume occupied in the blood, plays an effective role in detecting quantitative abnormalities. PCT correlates with PLT count and has comparable clinical significance.

### Clinical significance

The results of our cross-sectional study could have significant clinical implications for primary care if confirmed in longitudinal studies. Until now, there has been a diagnostic gap in the rapid and.

cost-effective identification of individuals with NWO. On the one hand, there is the useful yet simplistic BMI, which lacks precision in distinguishing between fat and lean tissue, often leading to underestimation of the NWO phenotype. On the other hand, there is the “gold standard” – DXA - which, due to its resource intensity and limited availability, poses significant challenges in primary care. Based on our findings, Complete Blood Count (CBC) testing shows potential as a preliminary, accessible, and cost-effective screening tool for early cardiometabolic risk assessment in individuals with a normal body weight. However, these results should be interpreted with caution; routine hematological parameters should be viewed as supplementary indicators that require further validation using advanced 5-part differential analysis and longitudinal observation to confirm their precise diagnostic utility in primary care settings.

### Limitations

The present study has several notable strengths. First, the participants were recruited from a well-characterized, homogeneous ethnic group. Second, the narrow age range (18–24 years) is a significant advantage, as it minimizes age-related variability and eliminates confounding factors associated with aging and co-morbidities that could otherwise skew the analysis. This homogeneity allows for a more precise evaluation of the specific impact of adiposity on hematological parameters in young, apparently healthy women. Furthermore, using this well-characterized cohort enabled a comprehensive analysis of hematological dimensions within a common physiological context, thereby enhancing internal validity and reducing physiological noise. To address potential selection bias, a rigorous 1:1 matching protocol was employed, and the high statistical power (0.951) confirms that the data reuse remains robust for identifying significant hematological deviations.

However, the authors are also aware of the study’s limitations. The primary methodological constraint is the use of a 3-part differential hematology analyzer. This technology provides a collective granulocyte count but lacks the resolution to specifically differentiate neutrophil subpopulations. Consequently, we were unable to calculate the neutrophil-to-lymphocyte ratio (NLR), a highly sensitive and clinically validated marker of systemic inflammation. This inability to pinpoint specific cellular drivers of the observed leukocytosis restricts the depth of the inflammatory profile provided in this study. Another limitation is the absence of multivariate regression analysis. Although we matched the groups 1:1 by age and BMI to ensure homogeneity and minimize the influence of these key variables, we cannot rule out the influence of other unmeasured confounders, such as physical activity levels or dietary patterns. Consequently, the observed differences in hematological parameters should be interpreted as associations rather than direct causal effects of increased adiposity. Furthermore, the cross-sectional design precludes the determination of causal relationships between adipose tissue distribution and the observed hematological alterations. Longitudinal studies are required to confirm these findings and establish the directionality of the associations. Moreover, hs-CRP was measured at a single time point; given its biological variability, this may not fully reflect long-term inflammatory status. We also lacked data on serum IL-6 and other proinflammatory cytokines or adipokines, which would have allowed for direct verification of the specific pathways linking adipose tissue to the observed leukocytosis. Finally, although we excluded participants with hormonal disorders, the specific phase of the menstrual cycle was not standardized. This represents a significant limitation, as estrogen directly promotes polyploidization and maturation of megakaryocytes by activating the ERβ/GATA1/STAT1 signaling pathway. Furthermore, estrogen-induced GATA1 transcription increases NF-E2 expression, a key factor for proplatelet formation. Because platelets and their precursors express estrogen receptors (ERα and ERβ), hormonal fluctuations throughout the cycle influence many platelet characteristics. Studies have shown that these fluctuations correlate with changes in collagen adhesion and a higher affinity for fibrinogen in the luteal phase compared with the follicular phase^48,49^. Therefore, because estrogen fluctuations affect both platelet count and reactivity, future studies should closely consider the phase of the menstrual cycle to minimize this physiological variability.

### Future research directions

Future research should focus on several key areas to validate and expand upon these findings. First, prospective longitudinal studies are essential to evaluate whether the observed hematological alterations in young NWO women translate into a higher incidence of cardiovascular events or type 2 diabetes in later life. Second, future investigations should include a broader panel of bioactive mediators, specifically proinflammatory cytokines (e.g., IL-6, TNF-α) and adipokines, to confirm the molecular mechanisms linking adipose tissue accumulation with systemic leukocytosis. Third, to minimize physiological variability, future protocols should standardize blood sampling by menstrual cycle phase and utilize 5-part differential hematology analyzers to assess specific inflammatory indices, such as the.

neutrophil-to-lymphocyte ratio (NLR). Moreover, future investigations should also explore the specific associations between internal fat distribution patterns, such as visceral adiposity and the android-to-gynoid ratio, and hematological dynamics, further to refine the metabolic profile of the NWO phenotype. Finally, interventional studies are warranted to determine whether lifestyle modifications (diet or physical activity) aimed at reducing body fat can reverse these unfavorable hematological profiles in women with the NWO phenotype.

## Conclusion

In conclusion, this study provides evidence that the NWO phenotype in young, apparently healthy women is associated with early, unfavorable changes in hematological parameters. We identified a specific profile characterized by elevated markers of subclinical proinflammatory state (WBC, LYM) and thrombotic potential (PLT, PCT, and notably P-LCC), which significantly correlate with body fat percentage and lipid disturbances. These findings suggest that despite maintaining a normal body weight, women with excess adiposity exhibit a subclinical proinflammatory and prothrombotic state similar to that observed in overt obesity.

Clinically, these results challenge the sufficiency of BMI as a standalone diagnostic tool. Routine hematological analysis - particularly focusing on WBC and P-LCC - may serve as an accessible, cost-effective screening method to identify young women at increased cardiometabolic risk who would otherwise be overlooked. Early detection of these subtle hematological deviations offers a critical window for targeted lifestyle interventions to prevent the progression to symptomatic cardiovascular disease. However, it should be emphasized that, due to the cross-sectional nature of this study, these findings represent associations and do not establish definitive causal relationships between increased adiposity and the observed hematological alterations.

## Supplementary Information

Below is the link to the electronic supplementary material.

Supplementary Material 1

## Data Availability

The datasets used and/or analyzed during the current study are available from the corresponding author on reasonable request.
