# Supplementary material for: Hematological markers of proinflammatory and prothrombotic states in normal-weight obesity (NWO)
Source: Sci Rep. 2026 Apr 28;16:19508. doi: 10.1038/s41598-026-48634-9 (PMC13287719; doi:10.1038/s41598-026-48634-9)
Supplement: Supplementary file 1 — Supplementary Material 1 [file 41598_2026_48634_MOESM1_ESM.docx]

**Table S1. Body composition characteristics of the subjects.**

|  | **All**  **(n = 176)** | | **NW**  **(n = 88)** | | **NWO**  **(n = 88)** | | P-value |
| --- | --- | --- | --- | --- | --- | --- | --- |
|  | **median** | Q_25_–Q_75_ | **median** | Q_25_–Q_75_ | median | Q_25_–Q_75_ |  |
| FM [kg] | **21.7** | 18.5–24.9 | **18.4** | 16.7–20.6 | **24.7** | 22.6–26.3 | p < 0.0001 |
| FMI [kg/m^2^] | **7.8** | 6.6–8.9 | **6.6** | 6.1–7.1 | **8.7** | 8.4–9.4 | p < 0.0001 |
| AFM [kg] | **12** | 10.2–13.5 | **10.3** | 9.2–11.4 | **13.3** | 12.2–14.4 | p < 0.0001 |
| AFMI [kg/m^2^] | **4.3** | 3.7–4.8 | **3.7** | 3.3–4 | **4.8** | 4.4–5.1 | p < 0.0001 |
| TAR | **0.73** | 0.66–0.79 | **0.7** | 0.65–0.78 | **0.74** | 0.68–0.81 | 0.051 |
| FMR | **0.74** | 0.69–0.79 | **0.71** | 0.67–0.76 | **0.76** | 0.71–0.80 | p < 0.0001 |
| APF [%] | **30.8** | 27.5–35.6 | **27.6** | 23.8–30.1 | **35.6** | 32.7–38.1 | p < 0.0001 |
| GPF [%] | **40.6** | 37.8–42.9 | **37.7** | 35.1–39.3 | **42.9** | 41.6–44.5 | p < 0.0001 |
| A/G | **0.77** | 0.71–0.83 | **0.73** | 0.67–0.79 | **0.82** | 0.76–0.88 | p < 0.0001 |
| VFA [cm^2^] | **45.2** | 36.1–56.4 | **39** | 33.7–48.5 | **51.8** | 42.3–63.2 | p < 0.0001 |
| VFM [g] | **217.9** | 174–271.8 | **187.8** | 162.4–233.7 | **249.7** | 203.8–304.5 | p < 0.0001 |
| VFV [cm^3^] | **235.6** | 188.1–293.8 | **203.1** | 175.5–252.7 | **270** | 220.3–329.2 | p < 0.0001 |
| FFM [kg] | **37.9** | 35.7–41.3 | **38.8** | 36.4–41.7 | **37.3** | 35.3–40.6 | 0.084 |
| FFMI [kg/m^2^] | **13.6** | 13–14.3 | **13.9** | 12.9–14.4 | **13.5** | 13–14.2 | 0.374 |
| AFFM [kg] | **16.4** | 14.9–17.7 | **16.7** | 15.5–18.3 | **16.1** | 14.5–17.5 | 0.042 |
| AFFMI [kg/m^2^] | **5.8** | 5.5–6.2 | **5.9** | 5.6–6.3 | **5.8** | 5.5–6.1 | 0.082 |
| TLM [kg] | **40.3** | 37.7–43.5 | **41** | 38.54–44.1 | **39.5** | 37.5–43.2 | 0.073 |
| TLMI [kg/m^2^] | **14.4** | 13.8–15.1 | **14.6** | 13.8–15.2 | **14.3** | 13.8–15 | 0.271 |
| ALM [kg] | **17.5** | 15.9–18.9 | **17.9** | 16.5–19.5 | **17.2** | 15.6–18.7 | 0.038 |
| ALMI [kg/m^2^] | **6.2** | 5.9–6.6 | **6.3** | 6–6.7 | **6.2** | 5.8–6.5 | 0.059 |

**Legend:** FM – fat mass; FMI – fat mass index; AFM – appendicular fat mass; AFMI – appendicular fat mass ratio;
TAR - trunk-to-appendicular fat ratio; FMR – fat mass ratio; APF – android percent fat; GPF – gynoid percent fat; A/G – android percent fat to gynoid percent fat ratio; VFA – visceral fat area; VFM - visceral fat mass; VFV - visceral fat volume; FFM – fat-free mass; FFMI – fat-free mass index; AFFM – appendicular fat-free mass; AFFMI - appendicular fat-free mass index; TLM – total lean mass; TLMI – total lean mass index; ALM – appendicular lean mass; ALMI - appendicular lean mass index

**Table S2. Correlations between hematological parameters and percent body fat, lipid profile, and cardiometabolic risk indices.**

|  | PBF  [%] | TC  [mM] | HDL-C [mM] | LDL-C [mM] | Non-HDL [mM] | TG  [mM] | Glucose [mM] | Insulin [μlU/ml] | HOMA-IR | TG/HDL-C | TG/G | VAI | LAP | CMI |
| --- | --- | --- | --- | --- | --- | --- | --- | --- | --- | --- | --- | --- | --- | --- |
| WBC | 0.20* | 0.13 | 0.10 | 0.11 | 0.13 | 0.21* | 0.11 | -0.01 | 0.10 | 0.08 | 0.20* | 0.05 | 0.13 | 0.08 |
| RBC | 0.18* | -0.11 | -0.16 | -0.09 | -0.08 | -0.08 | -0.23* | -0.06 | -0.12 | 0.05 | -0.13 | 0.02 | -0.09 | 0.01 |
| HGB | 0.14 | 0.22* | 0.15 | 0.22* | 0.22* | 0.15 | 0.21* | -0.12 | 0.01 | 0.06 | 0.19* | 0.03 | 0.15 | 0.03 |
| HCT | 0.16 | 0.24** | 0.22* | 0.21* | 0.21* | 0.18* | 0.22* | -0.10 | 0.04 | 0.04 | 0.22* | 0.02 | 0.23* | 0.01 |
| MCV | 0.07 | 0.35** | 0.35*** | 0.31** | 0.31** | 0.26** | 0.46*** | -0.01 | 0.16 | 0.01 | 0.34*** | -0.01 | 0.28** | 0.00 |
| MCH | 0.06 | 0.31*** | 0.31*** | 0.30** | 0.29** | 0.20* | 0.43*** | -0.03 | 0.12 | -0.02 | 0.29** | -0.04 | 0.19* | -0.03 |
| MCHC | -0.01 | 0.00** | -0.06 | 0.05 | 0.04 | -0.04 | 0.05 | -0.01 | -0.01 | -0.01 | -0.02 | -0.04 | -0.14 | -0.03 |
| PLT | 0.16 | -0.06 | -0.12 | -0.07 | -0.05 | 0.09 | -0.10 | 0.08 | 0.04 | 0.16 | 0.04 | 0.14 | -0.01 | 0.17 |
| PCT | 0.25** | 0.27** | 0.22* | 0.24** | 0.25** | 0.28** | 0.27** | 0.12 | 0.22* | 0.09 | 0.29** | 0.05 | 0.20* | 0.09 |
| MPV | 0.05 | 0.49*** | 0.52*** | 0.42*** | 0.41*** | 0.25** | 0.56*** | 0.08 | 0.29** | -0.15 | 0.37*** | -0.16 | 0.33** | -0.14 |
| PDWs | 0.06 | 0.47*** | 0.51*** | 0.40*** | 0.40*** | 0.24** | 0.54*** | 0.11 | 0.31** | -0.16 | 0.35*** | -0.16 | 0.31** | -0.15 |
| PDWc | 0.08 | 0.40*** | 0.44*** | 0.34*** | 0.33*** | 0.21* | 0.46*** | 0.07 | 0.24** | -0.15 | 0.31** | -0.15 | 0.28** | -0.14 |
| RDWs | -0.05 | 0.39*** | 0.38*** | 0.32*** | 0.32*** | 0.22* | 0.49*** | 0.16 | 0.32*** | -0.06 | 0.33*** | -0.08 | 0.31** | -0.09 |
| RDWc | -0.12 | 0.09 | 0.07 | 0.04 | 0.04 | -0.03 | 0.07 | 0.16 | 0.18 | -0.10 | 0.01 | -0.11 | -0.07 | -0.12 |
| LYM | 0.21* | 0.27** | 0.25** | 0.24** | 0.24** | 0.22* | 0.14 | 0.02 | 0.08 | -0.02 | 0.20* | -0.09 | 0.05 | -0.06 |
| MID | 0.11 | -0.02 | 0.09 | -0.08 | -0.08 | -0.03 | 0.00 | -0.10 | -0.03 | -0.14 | 0.00 | -0.18 | -0.13 | -0.18 |
| GRA | 0.15 | 0.06 | 0.01 | 0.05 | 0.07 | 0.13 | 0.09 | -0.02 | 0.08 | 0.08 | 0.14 | 0.05 | 0.14 | 0.07 |
| LYM% | 0.14 | 0.21* | 0.20* | 0.22* | 0.20* | 0.12 | 0.09 | 0.07 | 0.05 | -0.03 | 0.10 | -0.09 | -0.05 | -0.09 |
| MID% | 0.03 | -0.10 | 0.03 | -0.17 | -0.18 | -0.12 | -0.05 | -0.05 | -0.03 | -0.17 | -0.10 | -0.18 | -0.16 | -0.19* |
| GRA% | -0.09 | -0.14 | -0.18* | -0.12 | -0.11 | -0.04 | -0.05 | -0.06 | -0.04 | 0.10 | -0.03 | 0.15 | 0.08 | 0.16 |
| P-LCC | 0.21* | 0.32*** | 0.30** | 0.27** | 0.28** | 0.29** | 0.32*** | 0.07 | 0.21* | 0.02 | 0.31** | -0.01 | 0.20* | 0.02 |
| P-LCR | 0.06 | 0.36*** | 0.41*** | 0.30** | 0.30** | 0.18* | 0.39*** | 0.00 | 0.17 | -0.15 | 0.26** | -0.13 | 0.27** | -0.12 |

**Legend:** PBF - percent body fat; TC – total cholesterol; HDL-C – high-density lipoprotein cholesterol; LDL-C – low-density lipoprotein cholesterol; Non-HDL – non-high-density lipoprotein cholesterol; TG – triglycerides; HOMA-IR - homeostatic model assessment for insulin resistance; TG/HDL-C – triglycerides to HDL-C ratio; TG/G – triglycerides to glucose ratio; VAI – visceral adiposity index; LAP – lipid accumulation product; CMI – cardiometabolic index; WBC - white blood cells; RBC - red blood cells; HGB – hemoglobin; HCT – hematocrit; MCV - mean corpuscular volume; MCH - mean corpuscular hemoglobin; MCHC - mean corpuscular hemoglobin concentration; PLT – platelets; PCT – plateletcrit; MPV - mean platelet volume; PDWs - platelet distribution width; PDWc - platelet distribution width; RDWs - red cell distribution width; RDWc - red cell distribution width; LYM – lymphocytes; MID - mid-sized cells; GRA – granulocytes;
LYM% - lymphocyte percentage; MID% - mid-sized cell percentage; GRA% - granulocyte percentage; P-LCC - platelet large cell count; P-LCR - platelet large cell ratio;
* p < 0.05, ** p < 0.01, *** p < 0.001
